# Co-cultivation of the marine sponge *Halichondria panicea* and its associated microorganisms

**DOI:** 10.1038/s41598-019-46904-3

**Published:** 2019-07-18

**Authors:** Stephen Knobloch, Ragnar Jóhannsson, Viggó Marteinsson

**Affiliations:** 1 Microbiology Group, Department of Research and Innovation, Matís ohf., 113 Reykjavik, Iceland; 2 Faculty of Life and Environmental Sciences, University of Iceland, 101 Reykjavík, Iceland; 3 Marine and Freshwater Research Institute, Hafrannsóknastofnun, 101 Reykjavik, Iceland; 4 Faculty of Food Science and Nutrition, University of Iceland, 101 Reykjavik, Iceland

**Keywords:** Applied microbiology, Environmental microbiology

## Abstract

Marine sponges host bacterial symbionts with biotechnological potential, yet isolation of true sponge symbionts remains difficult due to their host dependency. Moreover, attempts to grow sponges for their pharmacologically-active compounds outside of their habitat often results in a shift of their microbial community. In this study we evaluate suitable sponge cultivation methods that allow maintenance of both the marine sponge *Halichondria panicea* and its associated bacteria in an *ex situ* environment. In addition, we present a method for co-cultivation of sponge explants and microbes separated by a membrane in a multi-chamber device. Tests on *ex situ* cultivation of *H. panicea* under different controlled conditions showed that only high water exchange rates in the aquarium enabled maintenance of its dominant symbiont “*Candidatus* Halichondribacter symbioticus” at a high relative abundance in the sponge body, a prerequisite for co-cultivation. The bacterial enrichment retrieved from co-cultivation contained bacteria from nine different classes in addition to sequences corresponding to “*Ca*. H. symbioticus”. This represents an increase of the cultivable bacterial classes from *H. panicea* compared to standard isolation techniques on solid media plates. The current study provides insights into sponge-microbe maintenance under *ex situ* conditions and proposes a new method for the isolation of sponge-associated bacteria.

## Introduction

Marine sponge (phylum *Porifera*) have been in the spotlight of marine derived natural product discovery for the past decades due to their rich inventory of pharmacologically-active compounds^1–3^. Despite this potential, sourcing sufficient quantities of a promising compound remains difficult due to the low concentrations of these chemicals in the sponge body. This issue, termed the “supply problem”, limits the advance of preclinical trials and hinders exploitation of sponges for other biotechnological purposes^4^. Apart from compounds derived from the sponge itself, it has been shown that sponge-associated microorganisms can be the source of bioactive compounds with pharmaceutically interesting properties^5,6^. Isolating these sponge-associated microbes could therefore circumvent the “supply problem”^7^. Sponges contain diverse and highly specific microbial communities which can contribute to functional attributes of the sponge holobiont^8^. These include obligate sponge symbionts as well as opportunistic and generalistic sponge-associated bacteria^9^.

The marine sponge *Halichondria panicea* has been used as a model sponge species for a variety of sponge physiological experiment^10–13^ and is well studied in terms of its biology and ecology in coastal areas^14–19^. Previous studies have found various bioactivities in extract of *H. panicea* and in microbes isolated from its body^20–25^, including extracts with cytotoxic properties^26,27^. *H. panicea* from various locations in the North Atlantic host the dominant bacterial symbiont “*Candidatus* Halichondribacter symbioticus” which often constitutes over half of the relative bacterial abundance in the sponge body^27–31^. Other bacteria within the sponge body vary depending on geographical location and time of sampling^27,29^. Despite variability they exhibit a clear difference in diversity from the surrounding seawater bacterial community, showing that, apart from “*Ca*. H. symbioticus”, *H. panicea* hosts other sponge-associated bacteria^28^. Bacterial isolation studies with *H. panicea* using standard cultivation methods have recovered strains associated with the taxa *Actinobacteria*, *Alpha*-, *Beta*- and *Gammaproteobacteria*, *Deinococcus*, *Cytophagia*, *Flavobacteria, Bacteroidetes* and low G + C Gram-positive bacteria^29,32^, including many *Actinobacteria* with bioactive properties^33,34^. To date, the dominant symbiont “*Ca*. H. symbioticus” has not been cultivated outside of its hosts. Being able to cultivate obligate sponge symbionts outside of, but in close proximity to, their host could allow valuable possibilities to study the mechanisms that lead to their host-dependency. Increasing the range of cultivable sponge-associated bacteria in general would be beneficial for biotechnological applications.

It is estimated that less than 1% of the bacterial diversity on earth is cultivable using traditional cultivation methods^35^. This has spawned new isolation and cultivation techniques ranging from microdroplet cultivation^36^ to *in situ* cultivation using new devices such as the ichip^37^ and other diffusion growth chambers^38^. Diffusion growth chambers consist of membranes with pore sizes small enough to trap microorganisms inside a chamber while allowing sufficient diffusion of metabolites between the chamber and the outside environment. The chambers are then inoculated with single microbial cells or microbial suspensions and are deployed back into their natural environment, thus providing the complex environmental conditions needed for growth^39^. Such systems have been, most notably, applied for the isolation of soil microorganisms^37,40^, but have also been implanted in wild sponges, yielding novel so far uncultivated microorganisms^41^. Whereas this is a promising approach, higher throughput co-cultivation of sponges and microorganisms using growth diffusion chambers requires both the proximity to the sponge and easy accessibility to the experiment. E*x situ* cultivation of sponges provides easier access and, in addition, allows for a control of all environmental parameters^42^. However, sponges have been reported to undergo a shift in their microbial diversity when cultured under *ex situ* conditions^43^, potentially detrimental to the isolation of sponge-associated microorganisms through a co-cultivation approach.

In this study we evaluate suitable sponge cultivation methods which allow maintenance of both the sponge and its associated bacteria in an *ex situ* environment, and present a new sponge-bacteria co-cultivation method for isolation of sponge-associated bacteria which can be used under laboratory setting. We apply 16S rRNA gene amplicon sequencing along with standard plating techniques to analyse the bacterial diversity in *H. panicea* during cultivation and compare the enriched bacterial fraction from co-cultivation to the results from standard plating.

## Results

### *Ex situ* cultivation of Halichondria panicea

**Figure 1:**
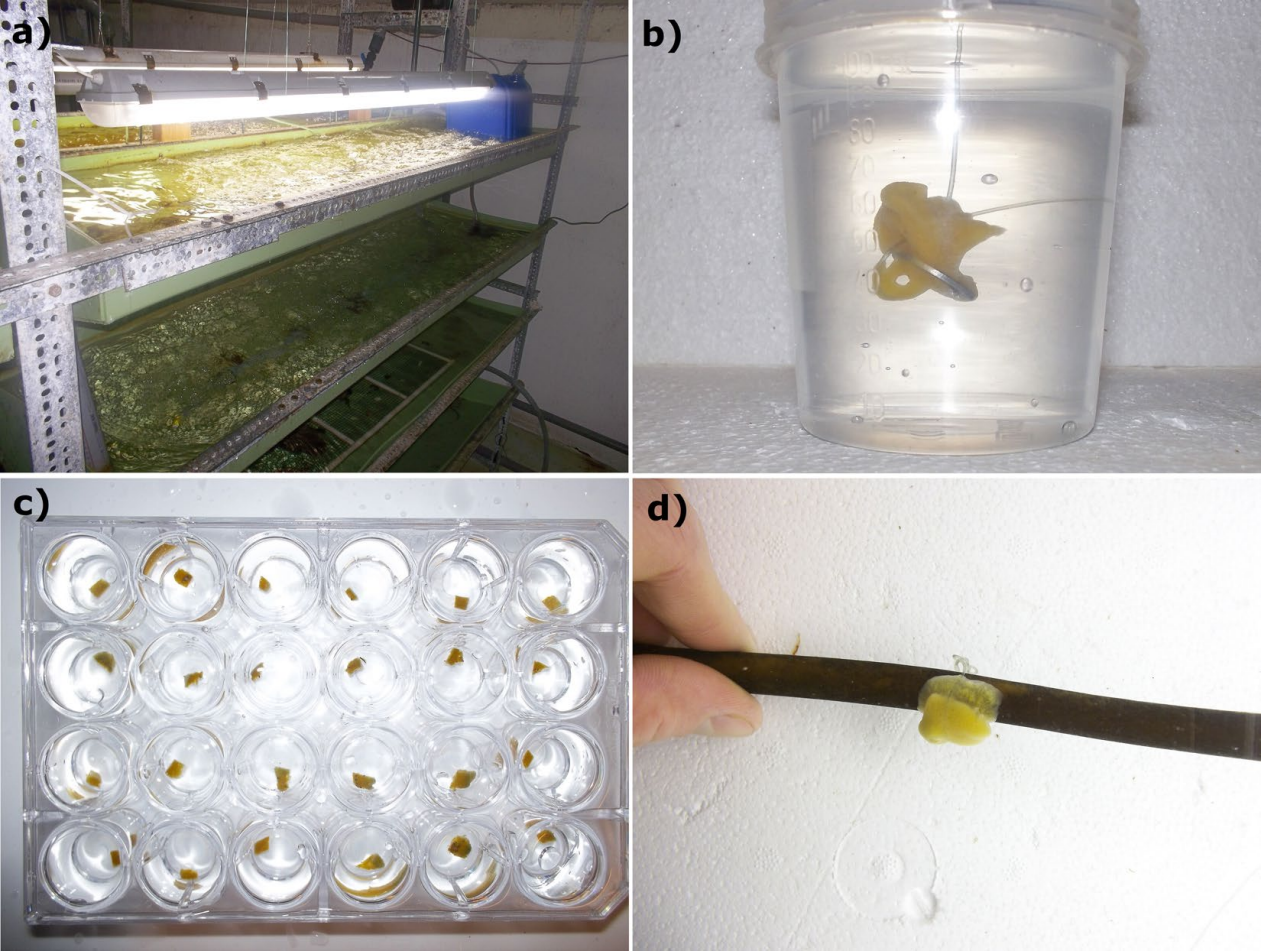
(**a**) Cultivation system for *H. panicea* in method 3 and 4. (**b**) Sponge explant attached to nylon string and metal washer in cultivation method 2. (**c**) Sponge explants distributed in 24-well plate before being deployed in aquarium in method 5. (**d**) Sponge explant attached to the thallus of *Laminaria* sp. in method 4.

To evaluate suitable methods for *ex situ* sponge cultivation five cultivation experiments were conducted with different aquarium set-ups (Fig. 1). Attachment to substrate, active pumping, growth as surface area expansion and survival were measured over the course of each experiment. Additionally, samples before and after each experiment were collected for microbial diversity analysis through 16S rRNA gene amplicon sequencing to determine shifts of the sponge-associated microbial diversity and the relative abundance of the dominant bacterial symbiont “*Ca*. Halichondribacter symbioticus”.

Attachment of explants to the substrate was visible within the first two weeks of the experiments, apart from in method 1 which used recirculated water and where over half of the explants showed no signs of attachment and eventually detached from the nylon string. Fastest attachment was visible in method 5 where all surviving explants attached to the surface within the first week of the experiment. Survival was above 50% in all experiments (Table 1) apart from method 1 where of the initial 9 explants 7 detached from the substrate or decreased largely in size, eventually leading to mortality. In method 2, 3, 4 and 5 survival was 63, 75, 100 and 63% of the explants or sponge individuals over the course of each experiment respectively.

**Table 1:** Description of methods tested for *ex situ* cultivation of *H. panicea*; Each experiment was carried out once over the duration indicated. NA: not applicable (sponges in method 3 were not removed from their original substrate); ^†^supplied from sand-filtered coastal seawater; *supplied from sand-filtered borehole seawater passed through a 3000 l public aquarium tank; +, positive; −, negative.

Method	Tank volume (l)	Feeding	Water exchange (tank volume d^−1^)	Duration	Number of sponge individuals	Substrate	Explant size	Attachment	Survival (>50%)
Method 1 (recirculating system)	60	yes	0	20 weeks	9	Nylon string	1 cm^3^	−	−
Method 2 (flow-through)	60	yes	10^†^	20 weeks	9	Nylon string	1 cm^3^	+	+
Method 3 (semi-recirculating)	360	no	2^†^	25 weeks	8	Rocks	NA	NA	+
Method 4 (semi-recirculating)	1360	no	4^†^	20 weeks	9	*Laminaria* sp.	1 cm^3^	+	+
Method 5 (flow-through)	15	no	720*	18 weeks	24	24-well plate (PS)	0.125 cm^3^	+	+

Within the first four weeks of all experiments, most sponges rearranged their aquiferous system and a single osculum, in the case of sponge explants, or multiple new oscula, in the case of sponge individuals kept on their original substrate, emerged and exhibited pumping activity. In method 5 oscula emerged earliest, within the first two weeks of the experiment, whereas in method 1 oscula on most explants were not observable. From all individuals used in the cultivation experiments growth, measured as an increase of the surface area, could only be observed in a single sponge individual in method 3. In most cases, sponge size did not vary between the beginning and end of the experiments and in some cases, especially in method 1, sponge size decreased during cultivation.

**Figure 2:**
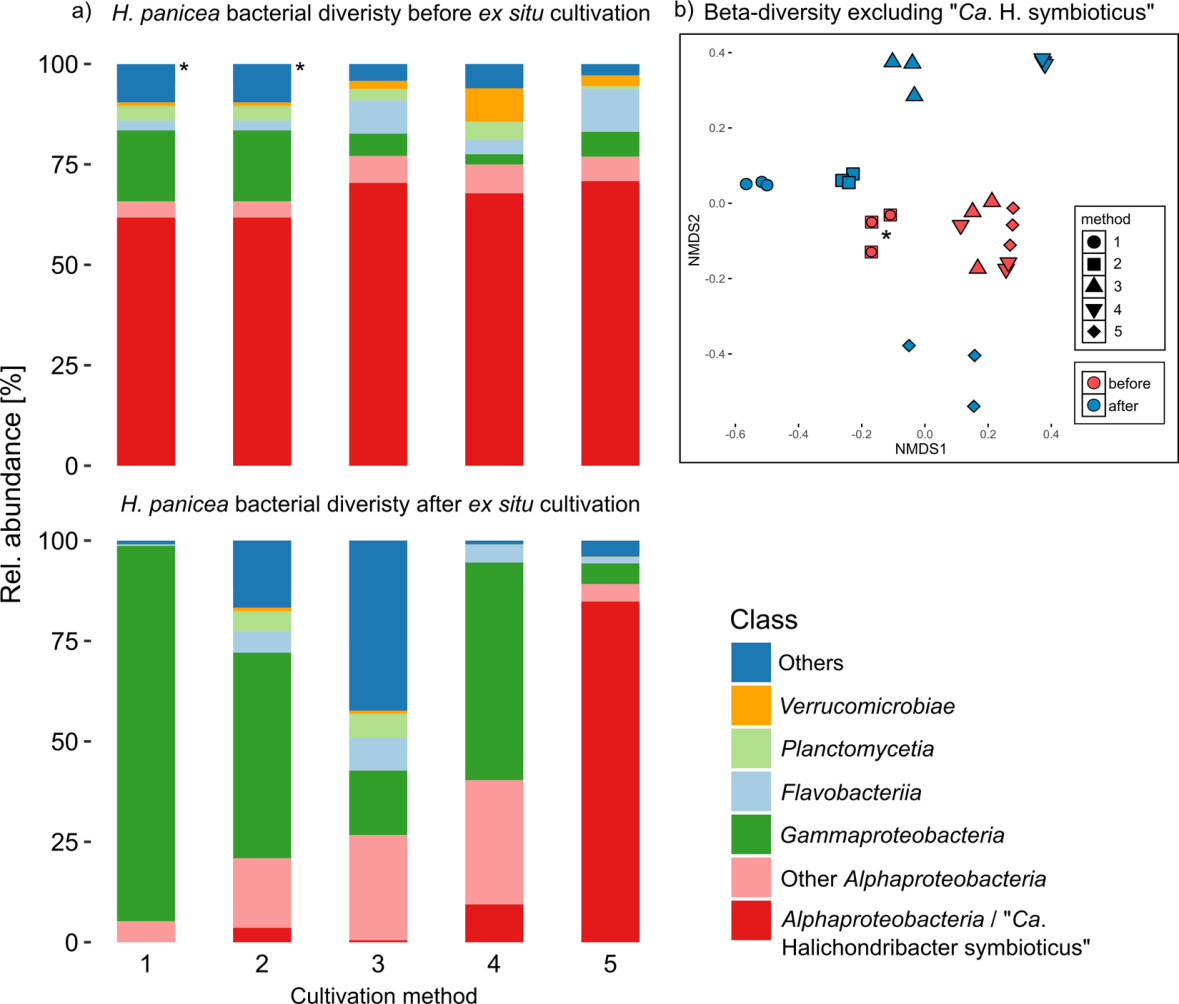
(**a**) Relative abundance of the bacterial diversity in *H. panicea* before and after *ex situ* cultivation using five different cultivation methods. Each bar represents the mean relative abundance of three sponge samples or explants. Classes representing less than 0.7% of the relative read abundance in the total dataset and unclassified taxa are grouped as “Others”. (**b**) NMDS ordination plot of Bray-Curtis dissimilarity matrix based on OTU-level distribution (stress value = 0.168). The same initial sponge was used for method 1 and 2, therefore sample sets marked with an asterisk are identical. Time between sample sets in weeks: 20 (method 1), 20 (method 2), 25 (method 3), 20 (method 4) and 18 (method 5).

**Figure 3:**
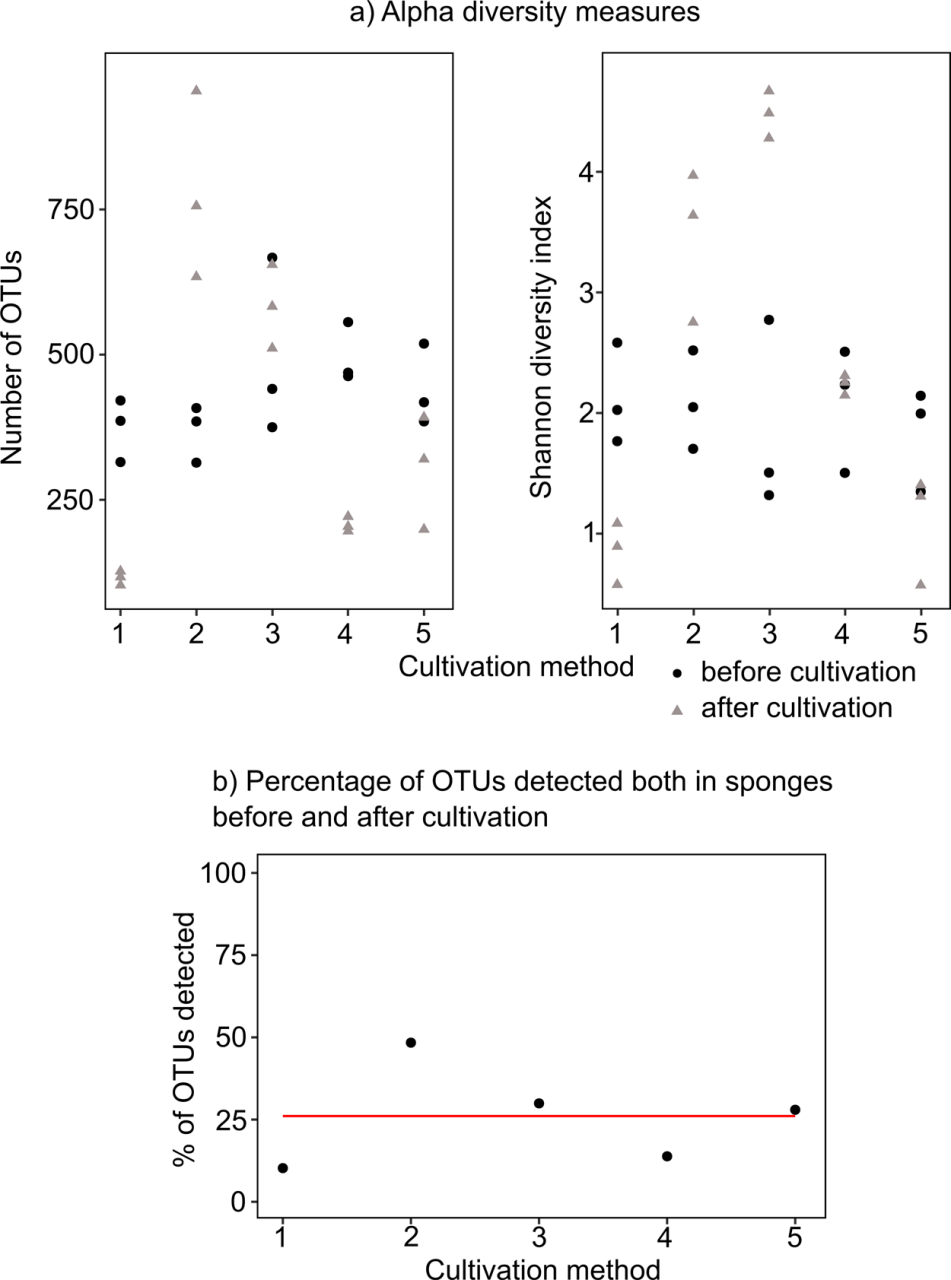
(**a**) Number of observed OTUs and Shannon diversity index of sponge samples before and after cultivation by method. (**b**) Percentage of OTUs in sponges before cultivation also detected in sponges after cultivation by method. Red line represents the average value across all methods.

The bacterial community of wild *H. panicea* collected for all experiments contained taxa predominantly assigned to the classes *Alphaproteobacteria*, *Gammaproteobacteria*, *Flavobacteriia*, *Planctomycetia* and *Verrucomicrobiae* (Fig. 2) and, to a lesser degree, to 28 other bacterial and 4 archaeal classes. In addition, the dominant symbiont “*Ca*. H. symbioticus” made up over 60% of the relative abundance in all samples. Although most sponges in methods 2 to 5 continued pumping and maintained their size over the course of the experiments, the bacterial diversity showed a marked shift between the beginning and end of the experiments (Fig. 2). The dominant symbiont “*Ca*. H. symbioticus” was reduced to less than 10% in samples collected in methods 1 to 4. In method 1 the relative abundance of “*Ca*. H. symbioticus” was reduced to an average of 0.004% of the relative abundance in addition to an overall reduction of OTUs (Fig. 3). “*Ca*. H. symbioticus” only remained the dominant bacterial taxon in method 5 with an average of 85% of the relative microbial abundance. After excluding “*Ca*. H. symbioticus” from the dataset the remaining bacterial diversity in the samples after cultivation appeared more dissimilar to the diversity of the sample before cultivation of each respective method (Fig. 2). In line with this finding only an average of 26% of the OTUs detected in the sponges before cultivation were also detected in the samples after the experiments (Fig. 3), showing that the majority of the initial bacterial taxa were lost during sponge cultivation. Despite this loss, the difference in alpha diversity in sponge samples before and after cultivation varied according to cultivation method used (Fig. 3). In methods 1 to 3 there was a significant (p < 0.05, t-test) increase or decrease of the alpha diversity, measured through Shannon diversity indices, before and after cultivation. In method 4 and 5 there were no significant differences (p > 0.1, t-test) in Shannon diversity indices despite a loss of detected OTUs in method 4 (Fig. 3).

### Bacterial diversity of sponge explants during co-cultivation

**Figure 4:**
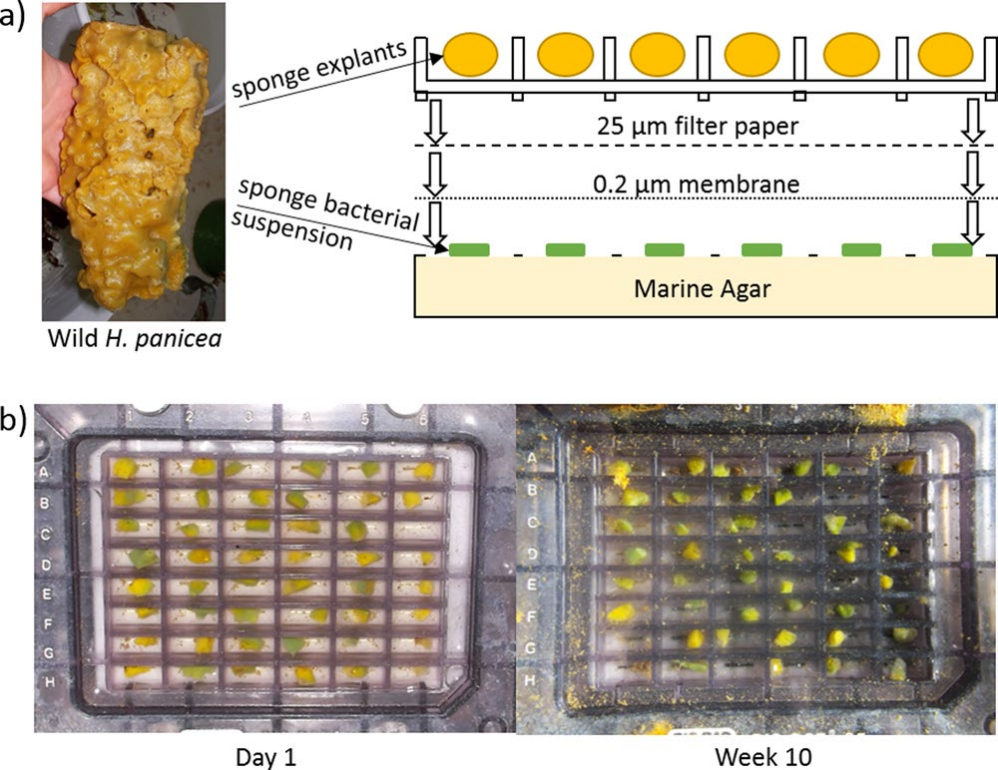
(**a**) Schematically diagram of sponge-bacteria co-cultivation set-up. The bottom chamber of a Bio-Dot SF microfiltration apparatus was filled with marine agar and each of the 48 chambers was inoculated with diluted bacterial suspension retrieved from a wild *H. panicea* specimen. These bacterial inoculations were separated from the top part of the apparatus through a 0.2 µm membrane and 25 µm filter paper. A sponge explant was placed into each of the 48 wells in the top of the apparatus, the device was clamped together and placed into a seawater aquarium for 10 weeks. (**b**) Sponge explants in co-cultivation device at day 1 and after week 10. Sponge explants collected for 16S rRNA gene amplicon sequencing and deceased explants were not replaced after the first week of the experiment. The co-cultivation experiment was conducted once during this study.

Based on the previous trials, method 5 was chosen for the sponge-bacteria co-cultivation experiment. *H. panicea* explants were cut and distributed into 48 wells of a Bio-Dot SF microfiltration apparatus (BioHit), separated by a 0.2 µm membrane from bacterial inoculations generated through serial dilutions from the same sponge individual (Fig. 4). Within the first week of deployment in the aquarium set-up detailed in method 5, *H. panicea* explants regenerated the cut surface and started attaching to the substrate of the co-cultivation device. Displaced explants or those showing signs of necrosis were replaced within the first week. During the following ten weeks four explants died and were not replaced. After ten weeks all remaining explants had developed at least one osculum which exhibited pumping activity when tested by placing food dye above the exhalent jet.

**Figure 5:**
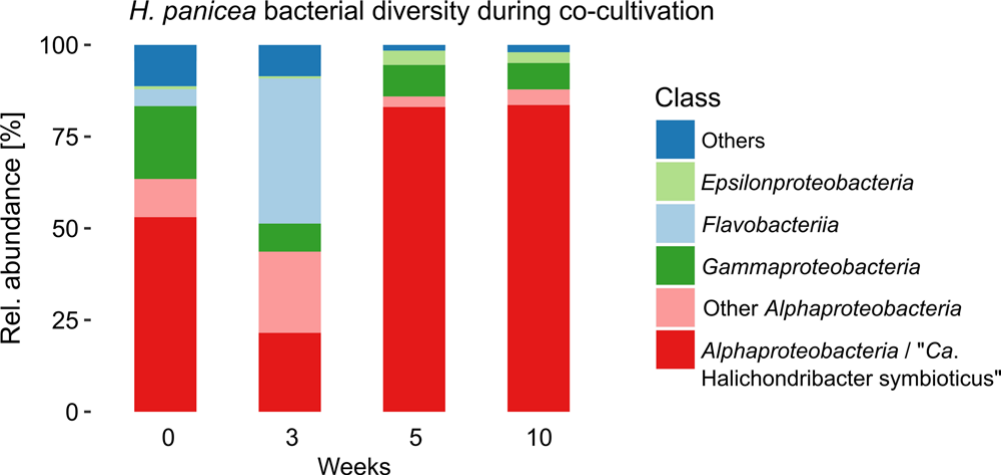
Relative abundance of the bacterial diversity in *H. panicea* at the start of the co-cultivation experiment and in *H. panicea* explants after 3, 5 and 10 weeks. Taxa are grouped by class level taxonomical association and classes representing less than 0.7% of the relative read abundance in the total dataset and unclassified taxa are grouped as “Others”.

**Figure 6:**
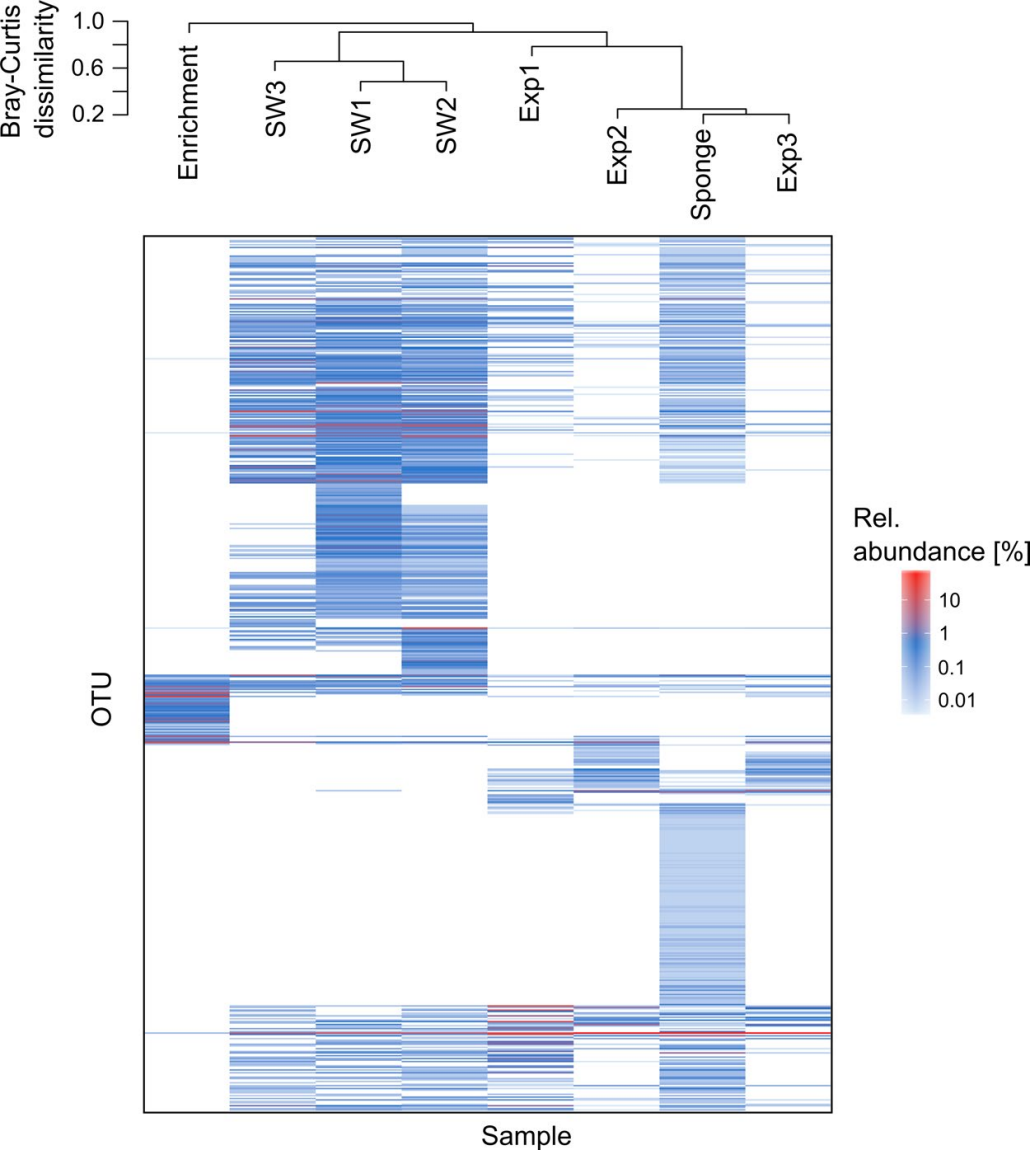
OTU heatmap showing the presence and relative abundance of OTUs In the original sponge sample used for the co-cultivation experiment (“Sponge”), the sponge explants (“Exp”), seawater samples from the co-cultivation aquarium (“SW”) and the bacterial enrichment pellet (“Enrichment”); Samples were ordered by hierarchical clustering of Bray-Curtis dissimilarities and method “average” of the hclust function in R.

A sample from the original sponge used for bacterial inoculation as well as three healthy looking explants, removed after three, five and ten weeks, were subjected to 16S rRNA gene amplicon sequencing. The dominant bacterial taxon, corresponding to the symbiont “*Ca*. H. symbioticus”, represented approximately 53% of the relative abundance in the sponge collected from the wild at the beginning of the experiment (Fig. 5). After three weeks the relative abundance of “*Ca*. H. symbioticus” had dropped to 22% of the relative abundance, whereas OTUs assigned to the class *Flavobacteriia* and other *Alphaproteobacteria* were more abundant than at day 0. In the explants analysed after five and ten weeks the relative abundance of “*Ca*. H. symbioticus” had increased to 83% and 84% of the relative abundance respectively. Similar to the previous experiments, 67% of the OTUs found in the sponge body before the start of the experiment were not detected in the explants anymore. Despite this shift in diversity, the microbial community in the explants was more similar to the original sponge sample than to the water samples collected from the tank at the same times, shown through hierarchical clustering of Bray-Curtis dissimilarities (Fig. 6).

### Comparison of bacterial diversity between co-cultivation enrichment and traditional isolation

**Figure 7:**
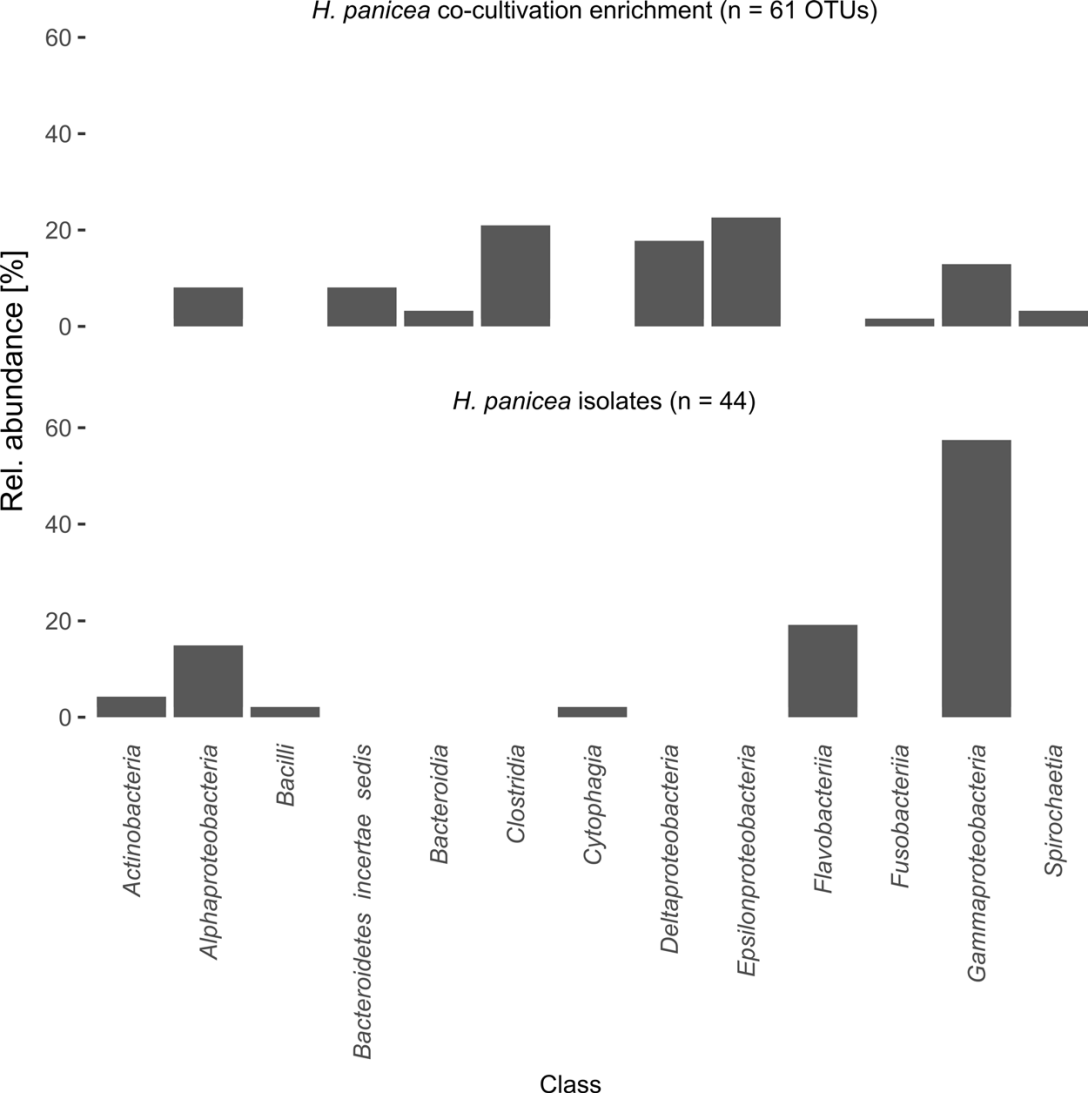
Relative abundance of OTUs enriched through sponge-bacterial co-cultivation (top) and strains isolated through standard plating method (bottom) by class level assignment.

After ten weeks the co-cultivation chamber was removed from the aquarium and the bacterial enrichment in form of visible pellets on the membrane was collected, pooled and subjected to 16S rRNA gene amplicon sequencing. The enrichment contained 61 OTUs in nine different bacterial classes, most of which assigned to the *Proteobacteria* (*Alpha*-, *Gamma*-, *Delta*-, *Epsilon*-) and *Clostridia* (Fig. 7 and see Supplementary Fig. S1 for phylogenetic tree). Only 14 OTUs in the enrichment were also detected in the original sponge used for bacterial inoculation (Fig. 6 and see Supplementary Table S1 for list of enrichment OTUs). In addition, these were mainly low abundant bacteria with less than 1% of the read abundance in the original sponge, apart from “*Ca*. H. symbioticus”, designated Otu1, of which eight reads were detected in the enrichment. A comparison against the NCBI nr/nt database showed that 80% of the OTUs showed closest sequence similarity to samples from marine origin and 33% to samples from marine invertebrate.

Using the traditional plating method, 253 colonies were isolated and screened using a MALDI-TOF biotyper. Out of these, 44 isolates showed a peptide mass fingerprint with sufficient dissimilarity to be classified as unique species and were selected for sequencing of the 16S rRNA gene. The Isolates were assigned to six different classes, whereas the majority of isolates belonged to the *Gammaproteobacteria* (Fig. 7 and see Supplementary Fig. S1 for phylogenetic tree). Similar to the co-cultivation enrichment, only 10 isolates showed high sequence similarity (>97.0%) to OTUs from the original inoculant and these were mostly low diversity OTUs (see Supplementary Table S1 for list of isolates). 84% of all isolates showed highest sequence similarity to samples isolated from marine habitats in the NCBI nr/nt database, and 34% were from marine invertebrates. In addition, two isolates showed highest sequence similarity to sequences previously isolated from *H. panicea*.

## Discussion

Five methods for *ex situ* cultivation of the sponge *H. panicea* were tested of which four showed promising results for maintaining *H. panicea* explants over the course of several weeks. The only method which was not suitable due to high mortality was the recirculating system in method 1. Although feed was provided through two microalgae strains and toxic build-up of ammonia and nitrite was prevented through biofiltration, the size of the sponge explants decreased over time and high mortality occurred. Previous studies have shown that *H. panicea* can utilise cells such as the microalgae *Rhodomonas* sp. as feed source with high efficiency^13,44^. The microalgae provided as feed in this study were in a similar size range to *Rhodomonas* sp. and could thus be regarded as suitable feed for the sponge. However, in regards to the carbon requirements of *H. panicea*^45^, insufficient feeding might have led to a decrease of explant size and eventually mortality. Further research needs to be conducted to evaluate suitable conditions for long-term cultivation of *H. panicea* in fully recirculating systems.

Despite successful maintenance of *H. panicea* using the other cultivation methods, only method 5 was suitable to maintain both the sponge and its dominant symbiont at a high relative abundance within the sponge body. The main difference between this method and the other methods was the high rate of water exchange within the tank, the water source and the size of the explants. As the water in method 5 was derived from the outlet of a public aquarium display tank and was supplied at a high rate compared to the sponge cultivation tank volume and the explant sizes, it is conceivable that enough feed was indirectly provided through the public aquarium to sufficiently nurture the sponge which, in turn, had a positive effect on the abundance of its symbiont. This is consistent with previous reports that report frequent detection of sponges, often unintentionally selected, in public aquaria, proving that they provide suitable cultivation conditions^4,46^. Conversely, a lack of feed provided by the other methods might have sustained the sponge over the cultivation period but triggered a reduction of its main symbiont. Alternatively, a build-up of undesirable metabolites in the sponge body and aquarium water due to a low water exchange rate in methods 1 to 4 could have prevented the proliferation of “*Ca*. H. symbioticus”.

Apart from “*Ca*. H. symbioticus”, the remaining microbial community in the sponge body underwent a shift in its composition with a loss of most of the original bacterial taxa at the end of the cultivation period, regardless of the method applied. It has previously been reported that sponges kept under *ex situ* conditions undergo a shift in their microbial community^47–49^ showing that changes in environmental conditions can have an impact on the sponge-associated microbial community. These changes could make the sponge habitat less suitable for opportunistic bacterial taxa otherwise associated with sponges in the wild. In addition, transient seawater bacteria detected in wild sponges might not be present under *ex situ* cultivation. Previous experiments on the bacterial diversity of *H. panicea* have shown that even in the wild its bacterial diversity is highly variable depending on site and time of the year^27–29^, indicating that many of the bacterial taxa routinely found in the sponge are transient. Nevertheless, up to 48% of the bacterial OTUs present within the original sponges were also present after cultivation in this study (Fig. 3), showing that some of the sponge-associated bacteria were retained at least over the course of several weeks.

During the co-cultivation experiment and using the sponge cultivation set-up described in method 5 we could revalidate the successful maintenance of the sponge and its dominant symbiont within the sponge body. However, an initial decline in the relative abundance of “*Ca*. H. symbioticus” was measured after 3 weeks before increasing back to a higher relative abundance similar that in its wild counterpart. This temporary decrease in symbiont abundance could indicate that the sponge explants were still adjusting to the new environment. This could be linked, as suggested above, to a reduced potential to feed while for instance rearranging the aquiferous system and regenerating the cut ectosome.

Using the novel co-cultivation method described in this study it was possible to enrich bacteria from a higher number of bacterial classes than using standard plate cultivation. Although isolation studies on *H. panicea* have previously been conducted using standard plating techniques^29,32,33^, this is the first time that bacteria from the classes *Spirochaetia*, *Fusobacteriia*, *Delta*- and *Epsilonproteobacteria*, and *Clostridia* were enriched from *H. panicea*. The occurrence of reads associated with “*Ca*. H symbioticus” in the enrichment is intriguing, however it is not excludable that they represent remaining DNA from the inoculation due to the high abundance of the symbiont in the inoculum. However, they could also represent cells of “*Ca*. H. symbioticus” that survived or grew outside of their hosts during co-cultivation. In the latter case further research on the proposed co-cultivation method are highly warranted as it could provide valuable insights into the symbiont’s physiology.

The fact that many of the enriched bacterial taxa and isolates were not detected within the original sponge used for inoculation, suggests that both methods also captured very low abundant taxa, possibly those present in seawater attached to the sponge or in its aquiferous system not detected in 16S rRNA gene amplicon sequencing of the sponge body. Though this could also be the effect of PCR bias or primer missmatch^50^. Despite this disparity, most taxa were similar to sequences previously isolated from marine habitats and marine invertebrate excluding contamination as a reason for this difference in detected and cultivated diversity.

## Conclusion

Novel approaches are needed to isolate so far uncultivated microorganisms for biotechnological purposes. Here we present a new sponge-microbe co-cultivation device using a diffusion growth chamber that can be deployed in a laboratory setting. Multiple wells allow for high throughput cultivation and re-isolation of microorganisms, while its *ex situ* deployment enables easy access to the device and close control over environmental parameters. We show that cultivation conditions have an influence on the sponge-associated microbial diversity and thus could impact co-cultivation success. Ensuring suitable conditions to maintain both the sponge and its associated microbes is therefore highly necessary. Whereas we were able to show a higher cultivation success using the co-cultivation method compared to standard isolation techniques, it must be noted that enrichment does not necessarily lead to isolation of microbes in pure culture and this would need to be investigated in future experiments.

## Methods

### Sponge collection

A total of 11 *H. panicea* individuals for cultivation, co-cultivation and bacterial isolation were collected from the intertidal and subtidal area in Seltjarnarnes (64°09′N 22°00′W) and Eyrarbakki (63°51′N 21°09′W) in South-West Iceland between April 2014 and June 2016. Samples were either cut from their substrate with a sterile scalpel or collected along with the substrate they were fastened to. Sponge individuals and samples were kept in 20 l buckets filled with seawater and were transported to the laboratory or cultivation facility within 1 hour after collection. Sponges were identified as *H. panicea* according to spicule structure and molecular markers as described in Knobloch *et al*.^28^.

### Sponge cultivation

For the sponge cultivation experiments *H. panicea* collected from the wild were cut into explants or kept on their original rock substrate. Explants were cut underwater with a sterile scalpel making sure to keep more than 50% of the explant surface covered with an intact ectosome to improve recovery. In total, five methods for cultivation of *H. panicea* were tested to find a suitable method for *ex situ* sponge maintenance (see Table 1). The first method consisted of a 60 l tank equipped with water aeration and commercial biofiltration mats. Water was circulated through the biofilter and within the tank using external pumps and airlift pumps respectively. Approximately 1 cm^3^ sized sponge explants were suspended into the water column from nylon stings with a metal washer attached to the bottom. 400 ml of mixed microalgae cultures at approximately 10^5^ cells ml^−1^ consisting of roughly equal concentrations of live *Dunaliella* sp. and *Phaeodactylum tricornutum* were added to the tank twice per week. Debris was removed from the tank twice a week using a syphon and seawater was replaced as necessary. Water loss from evaporation was replaced with deionised water. The second method was identical to the first method apart from that seawater was continuously added to the tank at a rate of approximately 600 l per day. The third method consisted of a tank set-up with a custom built bacterial moving bed biofilter, aeration for water movement and oxygenation, and artificial lighting set to 12 h dark and 12 h light (Fig. 1). Water was continuously supplied to replace the system volume of 360 l twice per day. Sponge individuals attached to stones collected in the intertidal area were placed onto a plastic mesh raised above the tank bottom. Additional feed was not supplied to this system. The fourth method consisted of the same specifications as method 3 apart from that a 1000 l tank was added to the system in which *Laminaria* sp. with an intact holdfast attached to a stone were placed. Approximately 1 cm^3^ sponge explants were attached to the *Laminaria* sp. thallus with nylon string. Artificial lighting set to 12 h dark and 12 h light was placed above the tank providing a light intensity of 15 klux at the water surface to maintain the seaweed. Water exchange rates in this method were adjusted to approximately four time the water volume per day. In the fifth method effluent water from a public aquarium was supplied to a 15 l tank. Approximately 0.125 cm^3^ sized sponge explants were placed individually into wells of a 24-well flat bottom TC-treated cell culture plate (Falcon). Aeration provided water movement and seawater was continuously exchanged at a rate of approximately 750 times the tank volume per day.

In methods 1–4 the water was supplied from a coastal seawater intake of a commercial aquaculture farm and was previously sand filtered to remove large particles from the water before entering the farm. The water used in method 5 was from a coastal seawater borehole and was also previously sand filtered before entering the public aquarium tanks. The water temperature in all system remained between 8 and 15 °C degrees throughout the experiments. Attachment of sponge explants was visually observed and by gently touching or tipping the substrate to see if explants were fastened. Sponges where necrosis was detected were removed from the tanks. Sponge explants which had fallen off the substrate or were displaced by other means were also removed from the tank. Pumping activity was measured by placing food grade dye above the osculum and observing the exhalent water current. Ammonia and nitrite concentrations in the tanks were monitored using commercial aquarium test kits from Seachem. The cultivation experiments lasted between 18 and 25 weeks (see Table 1). For microbial diversity analysis three sponge samples from separate individuals were collected before and after each experiment. All sponge samples were stored at −80 °C until proceeding with DNA extraction.

### Sponge-bacteria co-cultivation experiment

For the sponge-bacteria co-cultivation experiment a wild sponge was cut into ca. 0.125 cm^3^ explants which were distributed individually into the wells of a Bio-Dot SF microfiltration apparatus (BioHit). A bacterial inoculum was made by rinsing 10 g of the same sponge with sterile artificial seawater (450 mM NaCl, 10 mM KCl, 9 mM CaCl_2_, 14 mM MgCl_2_, 8 mM MgSO_4_) to remove bacteria attached to the outer surface and grinding the sponge in sterile calcium-magnesium free seawater (450 mM NaCl, 10 mM KCl). The sponge suspension was centrifuged for 2 minutes at 500 x g to remove the majority of sponge cells and the supernatant was subjected to serial dilutions of up to 10^5^ in sterile artificial seawater. The bottom container of the microfiltration apparatus was filled with Marine Agar (BD Difco) under sterile conditions by inserting the medium through the effluent tube of the apparatus until the medium reached the top layer of the well separation grid. Then the effluent tube was closed and the media was left to solidify. 3 µl of the 10^5^ sponge-bacterial dilution was placed onto the solid medium of each well and covered by 0.2 µm nylon membrane filters (Whatman) followed by seven layers of 25 µm filter paper (Miracloth, Merk-Millipore) to prevent the membrane from clogging. The top half of the microfiltration apparatus containing the sponge explants was briefly removed from the water and tightly screwed onto the bottom half and placed back under the water surface. This co-cultivation device was placed into a 15 l tank and kept under conditions as described for method 5 above. Sponge explants displaced or showing signs of necrosis within the first week were replaced with explants kept in a reserve tank. Explants deceased or displaced after the first week were not replaced. Wells were cleaned regularly to prevent build-up of debris above the filters and membrane separating the explants and the bacterial inoculum.

After ten weeks the device was removed from the tank and transported back to the laboratory on ice. The outside of the device was rinsed thoroughly with sterile water and the top half was removed, being careful not to displace or contaminate the membrane. The bottom of the membrane and surface of the solid media were searched for colony growth which was combined to a single pellet in a microcentrifuge tube and stored at −80 °C until DNA extraction.

### Bacterial isolation

For bacterial isolation using the standard plating method the same sponge bacterial suspension as for the co-cultivation experiment was used. Dilutions at 10^4^ and 10^5^ were plated on Marine Agar and Starch Yeast-Extract Peptone Sea Water Agar (10 g potato starch, 4 g Bacto Yeast Extract, 2 g Bacto Peptone, 15 g Bacto Agar in 1 litre of 0.2 µm filtered seawater), both being rich general purpose media. All media were additionally prepared with *H. panicea* sponge extract, made from a sponge suspension filtered through a 0.2 µm filter and added to the media at 0.5% (v/v) after autoclaving.

Inoculated plates were incubated at 10 and 22 °C and colonies were picked and restreaked on Marine Agar plates at least three times to assure strains were isolates. Isolated strains were analysed on a Microflex MALDI-TOF mass spectrometer (Bruker) after formic acid extraction. In short, a loopfull of an isolated colony was diluted in 300 µl of ultra-pure water in a microcentrifuge tube. 900 µl of pure ethanol was added and the cell suspension vortexed for 1 min followed by centrifugation at 13.000 × g for 2 min. The supernatant was removed and the pellet was air dried, after which 30 µl of 70% formic acid was added and mixed with the pellet by pipetting up and down. 30 µl of 100% acetonitrile was added to the tube and mixed carefully followed by centrifugation at 13.000 × g for 2 min. 1 µl of the supernatant was placed on a stainless steel MALDI target and allowed to air dry. Once dry, the spot was overlaid with freshly prepared HCCA (Bruker) matrix solution and run on the MALDI-TOF using default settings. A main spectra profile dendrogram was created based on m/z spectra comparison and strains clustering into same groups at low distance equivalents were considered as the same species. For each species cluster, one isolate was selected for sequencing of the partial 16S rRNA gene for which a colony of the selected strains was subjected to DNA extraction using the MasterPure DNA Purification Kit (Epicentre) according to the manufacturer’s instructions. The partial 16S rRNA gene was amplified using the primer pair F27/R806 (5′-AGAGTTTGATCMTGGCTCAG-3′/5′-GGACTACVSGGGTATCTAAT-3) and sequenced on a 3730 DNA Analyser (Applied Biosystems, Hitachi). Sequences were trimmed to position 341 and 785 (relative to the *E. coli* 16S rRNA gene) using the bioinformatics software Geneious, to be able to compare against 16S rRNA gene amplicons, and taxonomically classified using the SINTAX algorithm^51^ against the SILVA v123 LTP database^52^.

### Microbial diversity analysis

DNA was extracted from sponge samples, seawater samples and the bacterial pellet retrieved from co-cultivation using the MasterPure DNA Purification Kit according to the manufacturer’s instructions, apart from that the sponge samples were first homogenised in laboratory grade Millipore water by passing them at least 10 times through a 90-gauge syringe. A region of the 16S rRNA gene covering the V3-V4 variable region was amplified using the universal prokaryotic primer pair S-D-Bact-0341-b-S-17 (5′-CCTACGGGNGGCWGCAG-3′)/S-D-Bact-0785-a-A-21 (5′-GACTACHVGGGTATCTAATCC-3′)^53^ and sequenced on a Illumina MiSeq desktop sequencer. PCR conditions, library preparation and sequencing were performed as described in Knobloch *et al*.^28^.

Raw sequences were demultiplex and 40 and 60 bps were trimmed off the forward and reverse sequences respectively using the fastx_trimmer command of the FASTX-Toolkit^54^. Trimmed reads were merged using the fastq_mergepairs command of the USEARCH package^55^ and sequences smaller or larger than 30 bp of the expected amplicon size were removed using the cutadapt tool^56^. Reads were further filtered using the -fastq_filter command with a maxee value of 1.0, dereplicated using the -fastx_uniques command and clustered into operational taxonomic units (OTUs) at 97% sequence identity using the -cluster_otus command of the UPARSE pipeline^57^. Singleton OTUs were removed. OTUs were taxonomically classified using the SINTAX algorithm against the SILVA v123 LTP database. The OTU and taxonomy tables were imported and subsequently analysed in the package phyloseq^58^ in the statistical software R^59^ implemented in RStudio^60^. For alpha and beta diversity analysis, the read number of all samples were normalised to an even depth of 15000 using the rarefy_even_depth command of the phyloseq package. Plots were created using ggplot2^61^.

## Supplementary information

Supplementary Figure S1

Supplementary Table S1

## Data Availability

Raw 16S rRNA gene amplicon reads are deposited in the Sequence Read Archive under BioProject ID PRJNA521872.
