# Supplementary material for: Co-cultivation of the marine sponge Halichondria panicea and its associated microorganisms
Source: Sci Rep. 2019 Jul 18;9:10403. doi: 10.1038/s41598-019-46904-3 (PMC6639338; doi:10.1038/s41598-019-46904-3)
Supplement: Supplementary file 1 — Supplementary Figure S1 [file 41598_2019_46904_MOESM1_ESM.docx]

**Co-cultivation of the marine sponge *Halichondria panicea* and its associated microorganisms**

Authors: Stephen Knobloch, Ragnar Jóhannsson, Viggó Marteinsson


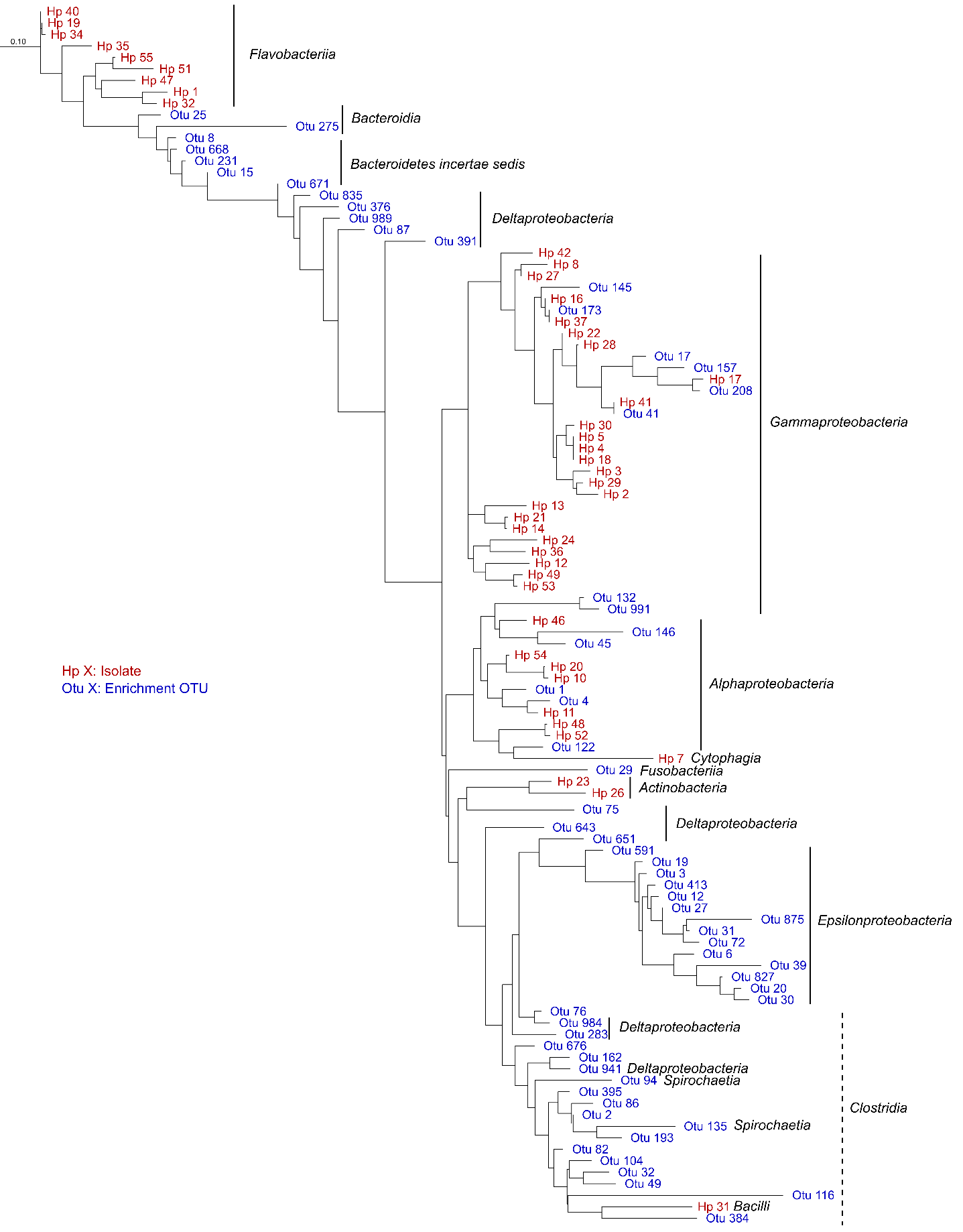


**Supplementary Figure S1** Maximum-likelihood phylogenetic tree of enriched OTUs and isolated strains based on their partial 16S rRNA sequence. Alignment and tree building was performed in ARB (Ludwig et al. 2004) using the PhyML algorithm (Guindon and Gascuel 2003)

Guindon S, Gascuel O (2003) A simple, fast, and accurate algorithm to estimate large phylogenies by maximum likelihood. Syst Biol 52:696–704

Ludwig W, Strunk O, Westram R, et al (2004) ARB: a software environment for sequence data. Nucleic Acids Res 32:1363–1371
