# Supplementary material for: Co-cultivation of the marine sponge Halichondria panicea and its associated microorganisms
Source: Sci Rep. 2019 Jul 18;9:10403. doi: 10.1038/s41598-019-46904-3 (PMC6639338; doi:10.1038/s41598-019-46904-3)
Supplement: Supplementary file 2 — Supplementary Table S1 [file 41598_2019_46904_MOESM2_ESM.docx]

**Co-cultivation of the marine sponge *Halichondria panicea* and its associated microorganisms**

Authors: Stephen Knobloch, Ragnar Jóhannsson, Viggó Marteinsson

**Supplementary Table S1**: List of bacterial isolates from *H. panicea* and enriched OTUs from the co-cultivation experiment.

|  | | **Assigned class** | | **Best hit (GenBank)** | | **Name (Genbank)** | | **Isolation source** | **Sequence identity (%)** | | **Rel. abundance *H. panicea* (%)** | | | **Rel. abundance in enrichment (%)** | |
| --- | --- | --- | --- | --- | --- | --- | --- | --- | --- | --- | --- | --- | --- | --- | --- |
| ***H. panicea* isolates** | | | | | | | | | | | | | | | |
|  | Hp_23 | | Actinobacteria | | MH701860.1 | | Micrococcus sp. strain CDR-SL-16 | | | Marine habitat | | 100 | 0 | | NA |
|  | Hp_26 | | Actinobacteria | | MH681545.1 | | Rhodococcus sp. strain SYP-A7263 | | | Unknown | | 100 | 0 | | NA |
|  | Hp_7 | | Cytophagia | | AB792995.1 | | Flammeovirga sp. KR07-01 | | | Marine reef | | 99.1 | 0 | | NA |
|  | Hp_1 | | Flavobacteriia | | JF827412.1 | | Uncultured bacterium clone 44F2 | | | Marine invertebrate | | 100 | 0.787 | | NA |
|  | Hp_19 | | Flavobacteriia | | AF493686.1 | | Flavobacteriaceae str. SW334 | | | Coastal seawater | | 99.1 | 0.014 | | NA |
|  | Hp_32 | | Flavobacteriia | | KM017080.1 | | Tenacibaculum sp. HMF2317 | | | Seawater | | 99.1 | 0.014 | | NA |
|  | Hp_34 | | Flavobacteriia | | EF491285.2 | | Uncultured Bacteroidetes bacterium clone S1-24 | | | Marine habitat | | 99.1 | 0.014 | | NA |
|  | Hp_35 | | Flavobacteriia | | MH594572.1 | | Uncultured bacterium clone RU12(10) | | | Marine algae | | 100 | 0 | | NA |
|  | Hp_40 | | Flavobacteriia | | AF493686.1 | | Flavobacteriaceae str. SW334 | | | Coastal seawater | | 99.4 | 0.014 | | NA |
|  | Hp_47 | | Flavobacteriia | | KX245373.2 | | Flagellimonas sp. strain ECD12 | | | Marine algae | | 100 | 0 | | NA |
|  | Hp_51 | | Flavobacteriia | | GQ274057.1 | | Uncultured Bacteroidetes bacterium clone 24D3 | | | Seawater | | 97.8 | 0 | | NA |
|  | Hp_55 | | Flavobacteriia | | JX530989.1 | | Uncultured Flavobacteriaceae bacterium clone C146300385 | | | Seawater | | 98.4 | 0 | | NA |
|  | Hp_31 | | Bacilli | | MG020099.1 | | Bacillus algicola | | | Marine invertebrate | | 100 | 0 | | NA |
|  | Hp_10 | | Alphaproteobacteria | | MF594171.1 | | Vibrio sp. strain 2216E-X-15 | | | Steel structure | | 100 | 0 | | NA |
|  | Hp_11 | | Alphaproteobacteria | | AY172307.1 | | Uncultured bacterium clone 38 | | | Unknown | | 99.7 | 0.009 | | NA |
|  | Hp_20 | | Alphaproteobacteria | | KP684473.1 | | Bacterium enrichment culture clone RB2LO08 | | | Halichondria panicea | | 100 | 0 | | NA |
|  | Hp_46 | | Alphaproteobacteria | | KX177742.1 | | Uncultured bacterium clone PW-15G12 | | | Seawater | | 100 | 0 | | NA |
|  | Hp_48 | | Alphaproteobacteria | | FR693315.1 | | Sphingopyxis sp. BB46 | | | Marine invertebrate | | 100 | 0.014 | | NA |
|  | Hp_52 | | Alphaproteobacteria | | MF461369.1 | | Sphingorhabdus sp. strain EL163 | | | Marine invertebrate | | 99.0 | 0.014 | | NA |
|  | Hp_54 | | Alphaproteobacteria | | KJ601444.1 | | Uncultured bacterium clone C34 | | | Coral tissue | | 100 | 0.009 | | NA |
|  | Hp_2 | | Gammaproteobacteria | | MG694250.1 | | Shewanellaceae bacterium strain PS03 | | | Beach sand | | 100 | 0 | | NA |
|  | Hp_3 | | Gammaproteobacteria | | MG283319.1 | | Shewanella halifaxensis strain LPB0184 | | | Sea sand | | 100 | 0 | | NA |
|  | Hp_4 | | Gammaproteobacteria | | KP684297.1 | | Bacterium DA91 | | | Halichondria panicea | | 100 | 0 | | NA |
|  | Hp_5 | | Gammaproteobacteria | | JX206721.1 | | Uncultured bacterium clone TV10-912_C10 | | | Marine sponge | | 99.1 | 0 | | NA |
|  | Hp_8 | | Gammaproteobacteria | | MF594182.1 | | Vibrio coralliilyticus strain 2216E-S-96 | | | Marine coral | | 100 | 0 | | NA |
|  | Hp_12 | | Gammaproteobacteria | | JN621578.1 | | Uncultured bacterium clone G13T5.7_B7 | | | Marine sediment | | 91.4 | 0 | | NA |
|  | Hp_13 | | Gammaproteobacteria | | MG254532.1 | | Pseudomonas fluorescens strain 8 | | | River | | 100 | 0 | | NA |
|  | Hp_14 | | Gammaproteobacteria | | MH057255.1 | | Microbulbifer sp. strain LSS-12 | | | Marine habitat | | 100 | 0 | | NA |
|  | Hp_16 | | Gammaproteobacteria | | MH512866.1 | | Vibrio sp. strain 101 | | | Sea water | | 100 | 0 | | NA |
|  | Hp_17 | | Gammaproteobacteria | | MG972754.1 | | Vibrio splendidus strain Vaa2 | | | Marine invertebrate | | 100 | 0 | | NA |
|  | Hp_18 | | Gammaproteobacteria | | JX206721.1 | | Uncultured bacterium clone TV10-912_C10 | | | Marine sponge | | 99.0 | 0 | | NA |
|  | Hp_21 | | Gammaproteobacteria | | KJ814573.1 | | Marine bacterium IIIA016 | | | Marine habitat | | 100 | 0 | | NA |
|  | Hp_22 | | Gammaproteobacteria | | KX453262.1 | | Pseudoalteromonas citrea strain hHt18-5 | | | Marine invertebrate | | 100 | 0 | | NA |
|  | Hp_24 | | Gammaproteobacteria | | MG815851.1 | | Halomonas subglaciescola strain KU-43 | | | Cheese brine | | 100 | 0 | | NA |
|  | Hp_27 | | Gammaproteobacteria | | KT582798.1 | | Uncultured Enterovibrio sp. clone 285KII | | | Marine fish | | 100 | 0 | | NA |
|  | Hp_28 | | Gammaproteobacteria | | MF594128.1 | | Pseudoalteromonas sp. strain 2216E-X-10 | | | Unknown | | 100 | 0 | | NA |
|  | Hp_29 | | Gammaproteobacteria | | JX436437.1 | | Shewanella sp. Wash4 | | | Marine sponge | | 100 | 0 | | NA |
|  | Hp_30 | | Gammaproteobacteria | | MG833271.1 | | Shewanella sp. strain 1719I3156 | | | Marine sponge | | 96.6 | 0.213 | | NA |
|  | Hp_36 | | Gammaproteobacteria | | KY655372.1 | | Spongiobacter sp. strain EA271 | | | Marine sponge | | 95.3 | 0 | | NA |
|  | Hp_37 | | Gammaproteobacteria | | MG996331.1 | | Uncultured bacterium clone Plate_K_H05_F | | | Seawater | | 100 | 0 | | NA |
|  | Hp_41 | | Gammaproteobacteria | | MG876115.1 | | Uncultured bacterium clone 4F_1112_20118_4586 | | | Seawater | | 100 | 0 | | NA |
|  | Hp_42 | | Gammaproteobacteria | | MF042743.1 | | Uncultured Idiomarina sp. clone 381 | | | Groundwater | | 100 | 0 | | NA |
|  | Hp_49 | | Gammaproteobacteria | | JX495001.1 | | Uncultured marine bacterium clone DLGF22 | | | Marine algae | | 100 | 0 | | NA |
|  | Hp_53 | | Gammaproteobacteria | | AF466927.1 | | Uncultured gamma proteobacterium isolate DGGE band D'7 | | | Seawater | | 100 | 0 | | NA |
|  |  | |  | |  | |  | | |  | |  |  | |  |
| **Co-cultivation enrichment OTUs** | | | | | | | | | | | | | | | |
|  | Otu_8 | | Bacteroidetes_incertae_sedis | | KX172965.1 | | Uncultured bacterium clone EzlYyy60 | | | Marine sediment | | 99.3 | 0 | | 0.007 |
|  | Otu_15 | | Bacteroidetes_incertae_sedis | | EU050907.1 | | Uncultured bacterium clone SS1_B_06_18 | | | Marine sediment | | 100 | 0.005 | | 9.043 |
|  | Otu_231 | | Bacteroidetes_incertae_sedis | | FJ716908.1 | | Uncultured bacterium clone B1_10.1_1 | | | Marine invertebrate | | 97.5 | 0 | | 0.082 |
|  | Otu_668 | | Bacteroidetes_incertae_sedis | | KX172965.1 | | Uncultured bacterium clone EzlYyy60 | | | Marine sediment | | 97.8 | 0 | | 0.004 |
|  | Otu_671 | | Bacteroidetes_incertae_sedis | | KM203417.1 | | Uncultured bacterium clone Plate5_4664_0_2 | | | Marine habitat | | 90.8 | 0 | | 0.019 |
|  | Otu_25 | | Bacteroidia | | FJ716944.1 | | Uncultured bacterium clone A4_10.4_1 | | | Marine sediment | | 96.2 | 0 | | 1.547 |
|  | Otu_275 | | Bacteroidia | | KU533822.1 | | Bacteroides sp.4SWWS3-28 | | | Marine sediment | | 99.3 | 0 | | 0.041 |
|  | Otu_376 | | Deltaproteobacteria | | KX088584.1 | | Uncultured bacterium clone MLGsedbac-8 | | | Marine habitat | | 88.6 | 0 | | 0.004 |
|  | Otu_835 | | Deltaproteobacteria | | KC606247.1 | | Uncultured bacterium clone CarbonSeq013_022210_A07 | | | Groundwater | | 88.1 | 0 | | 0.004 |
|  | Otu_2 | | Clostridia | | KF799151.1 | | Uncultured bacterium clone Woods-Hole_a5657 | | | Marine invertebrate | | 100 | 0.005 | | 32.099 |
|  | Otu_32 | | Clostridia | | JX391218.1 | | Uncultured bacterium clone H3078 | | | Marine sediment | | 100 | 0 | | 0.245 |
|  | Otu_49 | | Clostridia | | AJ441231.1 | | Uncultured firmicute | | | Marine invertebrate | | 99.8 | 0 | | 0.616 |
|  | Otu_76 | | Clostridia | | LN849485.1 | | Uncultured bacterium | | | Biogas reactor | | 91.1 | 0 | | 0.627 |
|  | Otu_82 | | Clostridia | | GU472396.1 | | Uncultured bacterium clone BBD-Feb09-6BB-70 | | | Coral tissue | | 96.7 | 0 | | 0.063 |
|  | Otu_86 | | Clostridia | | KF799151.1 | | Uncultured bacterium clone Woods-Hole_a5657 | | | Marine invertebrate | | 95.8 | 0 | | 0.434 |
|  | Otu_104 | | Clostridia | | KR086551.1 | | Uncultured bacterium clone DH162B34 | | | Marine habitat | | 100 | 0 | | 0.319 |
|  | Otu_116 | | Clostridia | | FJ223458.1 | | Uncultured bacterium clone 447 | | | Marine sediment | | 97.5 | 0 | | 0.156 |
|  | Otu_162 | | Clostridia | | AB704728.1 | | Uncultured bacterium M03-2-Bac-F6_27F_1_F06_038 | | | Borehole water | | 91.1 | 0 | | 0.200 |
|  | Otu_193 | | Clostridia | | KF323282.1 | | Uncultured bacterium clone GXTJ5A301BOFJ0 | | | Marine invertebrate | | 93.7 | 0 | | 0.293 |
|  | Otu_384 | | Clostridia | | MG367102.1 | | Uncultured Clostridiales Family IV bacterium clone TR_Beef_D2 | | | Marine sediment | | 99.1 | 0 | | 0.004 |
|  | Otu_395 | | Clostridia | | KF179748.1 | | Uncultured bacterium clone MAY9C13 | | | Coral tissue | | 96.9 | 0 | | 0.007 |
|  | Otu_676 | | Clostridia | | KF179748.1 | | Uncultured bacterium clone MAY9C13 | | | Coral tissue | | 92.5 | 0 | | 0.059 |
|  | Otu_29 | | Fusobacteriia | | KX956270.1 | | Uncultured bacterium clone OTU1955 | | | Marine sediment | | 99.8 | 0.009 | | 1.688 |
|  | Otu_1 | | Alphaproteobacteria | | KJ453525.1 | | Uncultured bacterium clone HP1-2.1 | | | Halichondria panicea | | 100 | 73.805 | | 0.030 |
|  | Otu_4 | | Alphaproteobacteria | | KX550187.1 | | Uncultured bacterium clone JCC_RecOTU_44 | | | Marine sediment | | 99.8 | 0.068 | | 0.004 |
|  | Otu_45 | | Alphaproteobacteria | | JN412112.1 | | Uncultured Bacteroidetes bacterium isolate DGGE gel band Chitin292_28 | | | Marine invertebrate | | 97.0 | 0 | | 0.638 |
|  | Otu_122 | | Alphaproteobacteria | | FJ175061.1 | | Uncultured bacterium clone sbrh_97 | | | Soil | | 100 | 0.005 | | 0.004 |
|  | Otu_146 | | Alphaproteobacteria | | EU458567.1 | | Uncultured bacterium clone HY2_g05_2 | | | Land animal | | 92.9 | 0 | | 0.100 |
|  | Otu_75 | | Deltaproteobacteria | | FJ202982.1 | | Uncultured bacterium clone SGUS569 | | | Coral tissue | | 97.2 | 0 | | 0.286 |
|  | Otu_87 | | Deltaproteobacteria | | KC606247.1 | | Uncultured bacterium clone CarbonSeq013_022210_A07 | | | Groundwater | | 92.2 | 0 | | 0.004 |
|  | Otu_283 | | Deltaproteobacteria | | HQ400926.1 | | Desulfovibrionaceae bacterium enrichment culture clone MS_OIL_O12 | | | Marine sediment | | 98.7 | 0 | | 0.119 |
|  | Otu_391 | | Deltaproteobacteria | | DQ088266.1 | | Uncultured bacterium clone cs49 | | | Sediment | | 91.8 | 0 | | 0.004 |
|  | Otu_643 | | Deltaproteobacteria | | HM598559.1 | | Uncultured bacterium clone Zeebrugge_B07 | | | Brakish sediment | | 92.3 | 0 | | 0.078 |
|  | Otu_651 | | Deltaproteobacteria | | GU061221.1 | | Uncultured delta proteobacterium clone 5m-23 | | | Seawater | | 90.1 | 0 | | 0.100 |
|  | Otu_941 | | Deltaproteobacteria | | KC607039.1 | | Uncultured bacterium clone CarbonSeq007_022210_E07 | | | Groundwater | | 92.0 | 0 | | 0.122 |
|  | Otu_984 | | Deltaproteobacteria | | EU236307.1 | | Uncultured bacterium clone Hg1a2D2 | | | Marine sponge | | 91.2 | 0 | | 0.278 |
|  | Otu_989 | | Deltaproteobacteria | | KC606247.1 | | Uncultured bacterium clone CarbonSeq013_022210_A07 | | | Groundwater | | 92.2 | 0 | | 0.019 |
|  | Otu_3 | | Epsilonproteobacteria | | JQ862033.1 | | Uncultured epsilon proteobacterium clone GC234-4-78 | | | Marine sponge | | 100 | 0.005 | | 27.698 |
|  | Otu_6 | | Epsilonproteobacteria | | KY236001.1 | | Uncultured bacterium clone HC6-92 | | | Marine habitat | | 100 | 0.009 | | 5.433 |
|  | Otu_12 | | Epsilonproteobacteria | | KP183006.1 | | Uncultured Arcobacter sp. clone 12S_65 | | | Seawater | | 99.8 | 0.023 | | 0.282 |
|  | Otu_19 | | Epsilonproteobacteria | | JF928745.1 | | Uncultured Arcobacter sp. clone OTU_A1_SP2_101 | | | Marine habitat | | 100 | 0.005 | | 2.364 |
|  | Otu_20 | | Epsilonproteobacteria | | FJ628231.1 | | Uncultured bacterium clone Nit2Au0637_410 | | | Seawater | | 99.8 | 0.005 | | 0.004 |
|  | Otu_27 | | Epsilonproteobacteria | | JX170296.1 | | Uncultured bacterium clone AJ-U-CD-188 | | | Marine invertebrate | | 100 | 0.014 | | 3.047 |
|  | Otu_30 | | Epsilonproteobacteria | | HQ203919.1 | | Uncultured bacterium clone SW-Apr-23 | | | Seawater | | 100 | 0.005 | | 0.152 |
|  | Otu_31 | | Epsilonproteobacteria | | LC133156.1 | | Uncultured Arcobacter sp. | | | Marine invertebrate | | 99.8 | 0.827 | | 0.200 |
|  | Otu_39 | | Epsilonproteobacteria | | FJ202693.1 | | Uncultured bacterium clone SGUS1281 | | | Coral tissue | | 97.4 | 0 | | 0.946 |
|  | Otu_72 | | Epsilonproteobacteria | | KY969129.1 | | Uncultured Arcobacter sp. clone D4 | | | Marine habitat | | 99.3 | 0.149 | | 0.148 |
|  | Otu_413 | | Epsilonproteobacteria | | EF029030.1 | | Uncultured bacterium clone root_Ell_39 | | | Seagrass | | 100 | 0 | | 0.404 |
|  | Otu_591 | | Epsilonproteobacteria | | KP994506.1 | | Uncultured Arcobacter sp. clone Pichicolo-G05 | | | Seawater | | 91.6 | 0 | | 0.004 |
|  | Otu_827 | | Epsilonproteobacteria | | AY768985.1 | | Uncultured bacterium clone FE2TopBac38 | | | Marine habitat | | 97.6 | 0 | | 0.004 |
|  | Otu_875 | | Epsilonproteobacteria | | FJ628346.1 | | Uncultured bacterium clone Nit5Au0628_769 | | | Seawater | | 90.6 | 0 | | 0.007 |
|  | Otu_17 | | Gammaproteobacteria | | KT341319.1 | | Uncultured bacterium clone a7-47 | | | Oil field | | 94.1 | 0 | | 6.086 |
|  | Otu_41 | | Gammaproteobacteria | | DQ357824.1 | | Moritella sp. SW43 clone 148.1 | | | Seawater | | 98.9 | 0 | | 1.180 |
|  | Otu_132 | | Gammaproteobacteria | | FJ618889.1 | | Uncultured bacterium clone BP5 | | | Marine invertebrate | | 97.2 | 0 | | 0.237 |
|  | Otu_145 | | Gammaproteobacteria | | KF325298.1 | | Uncultured bacterium clone G250WV301AHQ3C | | | Marine invertebrate | | 95.1 | 0 | | 0.393 |
|  | Otu_157 | | Gammaproteobacteria | | KT341319.1 | | Uncultured bacterium clone a7-47 | | | Oil field | | 93.2 | 0 | | 0.397 |
|  | Otu_173 | | Gammaproteobacteria | | EU617814.1 | | Uncultured bacterium clone B13S-47 | | | Marine sediment | | 100 | 0 | | 0.219 |
|  | Otu_208 | | Gammaproteobacteria | | KP952941.1 | | Uncultured bacterium clone I3Q1XXJ01AMV7I | | | Marine invertebrate | | 96.9 | 0 | | 0.442 |
|  | Otu_991 | | Gammaproteobacteria | | KF179764.1 | | Uncultured bacterium clone MAY9C35 | | | Coral tissue | | 98.3 | 0.005 | | 0.141 |
|  | Otu_94 | | Spirochaetia | | KC261847.1 | | Spirochaeta sp. K2 | | | Marine sediment | | 97 | 0 | | 0.590 |
|  | Otu_135 | | Spirochaetia | | KC261848.1 | | Spirochaeta sp. K1 | | | Marine sediment | | 89.4 | 0 | | 0.271 |
